# Multiple loci with cumulative effects on late maturity α-amylase (LMA) in wheat

**DOI:** 10.1007/s00425-023-04131-1

**Published:** 2023-04-11

**Authors:** Daryl Mares, Adinda Derkx, Diane E. Mather, Judy Cheong, Kolumbina Mrva

**Affiliations:** 1 School of Agriculture, Food and Wine, University of Adelaide, Waite Campus, Glen Osmond, SA 5064 Australia; 2 South Australian Research and Development Institute, Waite Precinct, Glen Osmond, SA 5064 Australia

**Keywords:** LMA phenotype, LMA resistance, Molecular markers, QTL

## Abstract

**Main conclusion:**

The cumulative action of combinations of alleles at several loci on the wheat genome is associated with different levels of resistance to late maturity α-amylase in bread wheat.

**Abstract:**

Resistance to late maturity α-amylase (LMA) in bread wheat (*Triticum aestivum* L.) involves a complex interaction between the genotype and the environment. Unfortunately, the incidence and severity of LMA expression is difficult to predict and once the trait has been triggered an unacceptably low falling number, high grain α-amylase may be the inevitable consequence. Wheat varieties with different levels of resistance to LMA have been identified but whilst some genetic loci have been reported, the mechanisms involved in resistance and the interaction between resistance loci requires further research. This investigation was focused on mapping resistance loci in populations derived by inter-crossing resistant wheat varieties or crossing resistant lines with a very susceptible line and then mapping quantitative trait loci. In addition to the previously reported locus on chromosome 7B for which a candidate gene has been proposed, loci were mapped on chromosomes 1B, 2A, 2B, 3A, 3B, 4A, 6A and 7D. These loci have limited effects on their own but have a cumulative effect in combination with each other. Further research will be required to determine the nature of the causal genes at these loci, to develop diagnostic markers and determine how the genes fit into the pathway that leads to the induction of *α-AMY1* transcription in the aleurone of developing wheat grains. Depending on the target environmental conditions, different combinations of alleles may be required to achieve a low risk of LMA expression.

**Supplementary Information:**

The online version contains supplementary material available at 10.1007/s00425-023-04131-1.

## Introduction

Late maturity α-amylase (LMA) can cause or contribute to low Falling Number and high grain α-amylase in wheat, depending on the genotype and the environmental conditions during ripening (Mares and Mrva 2014; Derkx and Mares 2020; Derkx et al. 2021; Liu et al. 2021a). Reduction of Falling Number below industry thresholds for receival of milling-grade wheat grain results in significant financial losses to growers. Despite uncertainty over whether LMA has serious effects on grain processing qualities (Newberry et al. 2018), Falling Number remains an internationally accepted measure of grain quality and an important specification for global grain trade. As a consequence, wheat breeders and the wheat industry in general have a vested interest in managing the risk of LMA.

Incidence of LMA has proven difficult to predict and, once LMA has been triggered, the effects on Falling Number cannot be reversed. Phenotyping methods for assessing potential to express LMA are costly. Realistically, phenotyping can only be applied towards the end of the breeding process, when the number of lines has been substantially reduced and the lines are nearing homogeneity. Lines that are found to have unacceptable risk of LMA may have to be discarded, despite having been carried through years of extensive evaluation and selection for other traits. This reduces capacity to achieve gains in other important traits. Genetic solutions, such as high-throughput molecular tools for marker-assisted selection that can be applied to large numbers of lines at an early stage of the breeding cycle, would be of considerable benefit in wheat breeding.

Wheat varieties with varying levels of resistance to LMA have been reported (Mares and Mrva 1993, 2008a; Mrva and Mares 2001a; Farrell and Kettlewell 2008; Mrva et al. 2009; Mohler et al. 2014; Derkx et al. 2021; Liu et al. 2021b). Quantitative trait loci (QTL) associated with resistance in some lines have also been reported (Mrva and Mares 2001a; Emebiri et al. 2010; Tan et al. 2010; Mohler et al. 2014; Derkx et al. 2021; Liu et al. 2021b). Whilst information on QTL locations and effects is useful in assessing the complexity of the genetic control of resistance, its broader application in practical wheat improvement is limited by a lack of knowledge of the genes involved.

Based on currently available evidence, it seems that several mechanisms may be involved in resistance and these mechanisms may be differentially affected by environmental conditions during grain ripening. One mechanism is associated with the semi-dwarfing alleles *RhtB1b* and *RhtD1b* (Mrva and Mares 1996). This mechanism, which appears to be particularly effective when wheat is ripened under warm temperatures (maximum daily temperatures > 25 °C), can be overcome by subjecting plants or spikes on tillers to a cool temperature shock during a sensitive stage in the middle of grain ripening (Mrva and Mares 2001b; Farrell and Kettlewell 2008; Mares and Mrva 2014; Derkx and Mares 2020) or by growing wheat under conditions where daily maximum temperatures are below around 23 °C (Derkx et al. 2021). This resistance is not evident in lines that carry alternate semi-dwarfing genes such as *Rht8* (Mrva et al. 2008) and is much less effective in combination with the 1B/1R translocation (Mrva et al. 2008; Farrell et al. 2013).

Differences in LMA resistance have also been observed among tall lines. A major QTL for this type of resistance has been mapped on the long arm of chromosome 7B (Mrva and Mares 2001a). Recently, a candidate gene, encoding an *ent*-Copalyl diphosphate synthase (CPS), was identified for this QTL (Derkx et al. 2021). Sequence variants were identified by sequencing the CPS gene from over 50 lines of diverse origin and LMA phenotype. Single nucleotide polymorphisms (SNPs) that distinguish among 9 CPS haplotypes (A through I) were then used to classify members of a large panel of varieties. Haplotypes A, B and C were shown to be associated with resistance while lines with haplotypes D through I exhibited a wide range of phenotypes, ranging from resistant to very susceptible within each haplotype group. While the resistance of Chinese Spring (haplotype A), Maringa (haplotype B) and Halberd (haplotype C) can be attributed to the 7B locus, the resistance of some other varieties, including Hartog (haplotype H) and Janz (haplotype F) must be conditioned by loci elsewhere in the genome. Janz is of particular interest since its resistance appears to be stronger and more stable across environments than that of Hartog.

The aims of this investigation were to assess the effectiveness of the 7B resistance alleles present in Halberd and Maringa, identify QTL associated with LMA resistance in Hartog and Janz and assess their effect on phenotype both singly and in combination with other QTL. These would be important first steps towards locating more genes involved in resistance, the development of diagnostic gene-based markers and unravelling the biochemical steps involved in triggering the transcription of the *α-Amy1* genes.

## Materials and methods

### Germplasm

Seed of three Australian varieties, Halberd (Scimitar/Kenya-C-6042//Bobin/3/Insignia-49, a tall LMA-resistant variety released in 1969), Janz (3-AG-3/4*Condor//Cook, a semi-dwarf LMA-resistant variety released in 1989), Hartog (Vicam-71//Ciano-67(sib)/Siete Cerros-66/3/Kalyansona/Bluebird, a semi-dwarf LMA-resistant variety released in 1982), the semi-dwarf breeding line RAC655 (CHA/Mengavi-8156//Ciano-67(sib)/Gallo/3/Bezostaya-2(CO-2224)/4/RAC-309-S, breeding line from the late 1990s not released commercially due to a high LMA rating) and the Brazilian variety Maringa (Frontana/Kenya-58//Ponta-Grossa-1, a tall LMA-resistant variety released in 1966), were obtained from stocks maintained at the University of Adelaide. These varieties had all maintained a consistent LMA phenotype over many experiments and seasons. They are all hard-grained spring wheat lines with no vernalisation or photoperiod requirement. All have white grain, except for Maringa which is a red-grained wheat. Hartog, Janz and RAC655 are included in the panel of LMA standards that is used in Australia to determine LMA risk, a process that is a prerequisite for grade classification and variety release.

### Populations

Single-seed descent was used to develop populations from the cross combinations Halberd (LMA-resistant)/Hartog (LMA-resistant) (145 lines), Maringa (LMA-resistant)/Hartog (LMA-resistant) (192 lines), RAC655 (LMA-susceptible)/Hartog (LMA-resistant) (327 lines) and Janz (LMA-resistant)/RAC655 (LMA-susceptible) (185 lines). Where one parent was a semi-dwarf or the parents carried different semi-dwarfing alleles, selection for tall plants was applied from F_2_ onwards to overcome the confounding effects of semi-dwarfing genes, *Rht-B1b* and *Rht-D1b*, and simplify phenotyping. For tall genotypes (those fixed for *Rht-B1a* and *Rht-D1a*), expression of the trait in the field and glasshouse environments is constitutive and does not require a cool temperature shock (Mares and Mrva 2008b). In the RAC655/Hartog population, selection for tall phenotype was not possible because both parents are fixed for the *Rht-D1b* semi-dwarfing allele.

### Plant growth conditions

LMA is a stochastic trait that is strongly influenced by the environment, in particular temperature. Grain α-amylase protein content can vary between plots, between individual plants, between spikes on individual plants and between grains in a spike (Mares and Mrva 2008a; Derkx and Mares 2020). Previous studies have reported the use of a cool temperature shock to trigger LMA in semi-dwarf varieties. Where the cool temperature shock treatment is used, plants or tillers must be transferred to the cool temperature environment during the sensitive phase in grain development (Derkx and Mares 2020) that has to be assessed visually. This phase corresponds to a thermal time of 650–800 °Cd, depending on the genotype. At this thermal time, the grains reach their maximum fresh and dry weights, a moisture content of 35–45%, begin to turn yellow in colour and start to lose moisture more rapidly (Derkx and Mares 2020). This corresponds to the soft dough stage (Zadoks growth stage 85) and the transition from the grain filling phase to the maturation and desiccation phase at physiological maturity. After this peak time point had been reached, the sensitivity for the cool-shock treatment declined again. The time for grains to reach this stage varies with temperature which determines the rate of grain development. In addition, wheat lines vary in their flowering time which inevitably means that the temperatures prior to the cool shock are not necessarily the same for all lines. To limit the variability, all but two phenotyping experiments involved plants grown in glasshouses with varying degrees of temperature control. Parental lines and populations were grown in multiple experiments in order to achieve the best possible estimate of LMA phenotype.

### Parental lines

The LMA phenotype of the semi-dwarf lines, RAC655, Hartog and Janz, was determined using two different protocols (Derkx and Mares 2020). Plants grown in a warm glasshouse and subjected to a cool temperature shock (12 experiments) or plants grown in a glasshouse where the daily maximum temperature was maintained below 23 °C (6 experiments), respectively. Experiments were conducted in both winter and summer. The warm glasshouse was equipped with evaporative air-conditioning but temperature varied due to differences in ambient temperature and daily solar radiation. Plants were grown in 9 cm square 17 cm deep pots with two plants per pot and two replicate pots per line. Replicates were positioned in rectangular, randomised blocks. Mean maximum temperatures in the warm glasshouse for the period anthesis to maturity were typically between 25 and 30 °C. For cool shock treatments, tillers were tagged at anthesis (first anthers extruded; Zadoks growth stage 61) and then transferred to a controlled environment room with temperatures set at 18 °C during the 14 h light period and 12 °C during the dark for 7 days (Derkx and Mares 2020). The correct time of transfer was determined visually as the point when grains just started to turn yellow. The timing varied slightly within the range 26–30 days post-anthesis (DPA) depending on temperature which influenced the rate of grain development. Following the cool temperature treatment, the tillers were transferred to a warm controlled environment room with temperatures set at 25 °C during the 14 h light period and 15 °C during the dark respectively to complete ripening. Plants remaining in the glasshouse were used as untreated controls.

The cooler glasshouse was equipped with heating, cooling and supplementary lighting (E602G 630 W Grow Lights, Heliospectra, Göteborg, Sweden). Temperatures were set at 22–23 °C day and 15 °C night on a diurnal cycle with a photoperiod of 14 h as described by Derkx and Mares (2020). Plants were harvested when ripe for phenotyping. The LMA phenotype of tall lines, Halberd and Maringa, was determined in both glasshouse environments but without a cool shock.

### Populations

Halberd/Hartog and Maringa/Hartog lines were initially grown in the field in 2013 as replicated twin rows 1 m in length. Plots were sampled for phenotyping at harvest-ripeness (grain moisture of around 12% FW) which was reached at 50–55 days post-anthesis (DPA). Halberd/Hartog, Maringa/Hartog and Janz/RAC655 lines were also grown in 4 (2014–2017), 5 (2014–2016) and 2 (2018 and 2019) experiments, respectively, in the cool glasshouse. Plants were grown in 9 cm square 17 cm deep pots with two plants per pot and two replicate pots per line. Plants were harvested when ripe for phenotyping.

The RAC655/Hartog semi-dwarf population, which required a cool temperature shock treatment for full expression of LMA phenotype, was grown in 5 experiments between 2016 and 2019 in 9 cm square 17 cm deep pots with two plants per pot and two replicate pots per line in the warm glasshouse. Plants were subjected to a cool temperature shock as described above for the parental lines.

### Determination of LMA phenotype as high pI α-amylase protein content

Grain was milled into a fine meal using a Perten LM-3310 laboratory burr mill equipped with type-2 fine grinding discs (Perten Instruments, Macquarie Park, NSW, Australia). Four 100 mg technical replicates per grain sample were assessed for high-pI α-amylase protein content using a sandwich ELISA assay developed by Verity et al. (1999) and the protocol described in Barrero et al. (2013) adapted to a 96-well format with C96 Maxisorb Nunc-Immuno microplates (Nunc A/S, Roskilde, Denmark). The second antibody used in the sandwich ELISA bound the high-pI α-amylase (Verity et al. 1999) but not low-pI α-amylase. This eliminated potential confounding effects due to any residual low pI α-amylase retained from early grain development. Spectrometric measurements were performed using a Benchmark™ Plus microplate reader (Bio-Rad Laboratories, Hercules, CA, USA) at 595 nm. Pure high-pI α-amylase protein was not available for use as a standard in the assay but a previous study by Derkx and Mares (2020) reported that high-pI α-amylase protein content measured as ELISA OD was strongly correlated with both total α-amylase activity and falling number. The high-pI α-amylase-specific ELISA has the advantages of being specific for the isozyme present in LMA-affected grain, is very sensitive and can be readily applied to analysis of large numbers of small grain samples. As the test protocol was identical for all the experiments in this study, the results were expressed in optical density (ELISA OD) units. Polyclonal and monoclonal antibodies required for LMA ELISA were covered by a sub-licence to the University of Adelaide by the Grains Research and Development Corporation, Australia, and were provided from stocks maintained by the South Australian Research and Development Institute, Urrbrae, South Australia. Labelled (horseradish peroxidase, HRP) anti-mouse antibody was sourced from Merck Australia, while the substrate for HRP, Blue Substrate ESBP1000, was obtained from ELISA Systems (Queensland, Australia). Other chemicals used in the study were obtained from Sigma-Aldrich Australia.

Data for individual lines from each of the populations were averaged across experiments with the aim of achieving the best estimate of phenotype.

### Genotyping

DNA was extracted from the youngest fully expanded leaf using a phenol/chloroform method (Rogowsky et al. 1991; Pallotta et al. 2000) and quantified using a NanoDrop 1000 spectrophotometer (Thermo Fischer Scientific, Wilmington, DE, USA). Halberd/Hartog was genotyped using the AWW525 marker, which amplifies a 762-bp product from the Halberd allele (*Bot-B5b*) of a boron tolerance gene on 7B and no product from the Hartog allele (*Bot(Df)-B5h)* of that gene (Pallotta et al. 2014) (Supplementary Figs. S1–S3).

Maringa/Hartog was genotyped using KASP assays for SNPs linked with the LMA QTL located on chromosome 7B reported by Derkx et al. (2021). Primer sequences used to genotype these populations are listed in Supplementary Table S1. The KASP assays were applied on an automated SNPLine system (LGC Limited, Teddington, UK), according to the manufacturer’s instructions. The RAC655/Hartog and Janz/RAC655 populations were genotyped by Diversity Arrays Technology (Bruce, ACT, Australia) using DArTseq genotyping-by-sequencing technology (www.diversityarrays.com/dart-application-dartseq). Genetic linkage maps were constructed for those populations in the R Statistical Computing Environment (R Core Team 2016) using the packages R/ASMap (Taylor and Butler 2016), which implements the MSTMap algorithm (Wu et al. 2008), and R/qtl (Broman et al. 2003). QTL analysis was conducted by simple interval mapping using R/qtl, with test statistic thresholds set using 10,000 permutations to achieve a genome-wide significance level of 0.05.

## Results

### LMA phenotypes of parental lines

Four LMA-resistant lines and an LMA-susceptible line, RAC655, were compared in glasshouse experiments under different environmental conditions (Table 1, Supplementary Table S2). The semi-dwarf LMA-susceptible line, RAC655, had an LMA-resistant phenotype when grain developed and ripened under warm temperatures (daily maximum temperatures > 25 °C), but showed a strong LMA response to a cool temperature shock or when grown under cool temperatures where daily maximum temperatures were maintained below 22–23 °C. High levels of high-pI α-amylase protein were synthesised and retained in the grain at harvest-ripeness. This LMA response pattern has been observed for many other commercial semi-dwarf wheat varieties (Derkx and Mares 2020; Derkx et al. 2021). By contrast, the semi-dwarf varieties Hartog and Janz generally did not respond to these LMA-inducing conditions. Significantly, Hartog and Janz have been placed in CPS haplotype groups (H and F, respectively) that include some very susceptible varieties (Derkx et al. 2021). The tall LMA-resistant varieties Halberd and Maringa maintained an LMA-resistant phenotype across environments but were not routinely subjected to a cool temperature shock, since previous research had indicated that tall lines are less sensitive to temperature variation and susceptible tall lines express LMA even when grown under warm to very warm conditions (Mares and Mrva 2014; Derkx et al. 2021).

**Table 1:** LMA phenotypes of four LMA-resistant lines and one LMA-susceptible line shown as means ± standard errors across experiments, with the numbers of experiments shown in brackets. The CPS haplotypes (H, F, C & B) reflect sequence variation in a CPS gene (Derkx et al. 2021) on chromosome 7B. High-pI α-amylase-specific ELISA values shown in bold were associated with a strong colour change in the assay and indicate that α-amylase protein was present. Values in plain font were not associated with a colour change and indicate that α-amylase protein was absent or below the level of detection

Line	LMA rating	CPS haplotype	LMA phenotype (high-pI α-amylase protein ELISA OD)		
			Untreated^a^	Cool temperature shock^b^	Cool temperature^c^
Hartog	Resistant	H	0.08 ± 0.01 (10)	0.14 ± 0.02 (12)	0.08 ± 0.01 (4)
Janz	Resistant	F	0.09 ± 0.01 (10)	0.13 ± 0.03 (12)	0.08 ± 0.01 (4)
Halberd	Resistant	C	0.07 ± 0.01 (4)		0.07 ± 0.01 (4)
Maringa	Resistant	B	0.08 ± 0.01 (7)		0.08 ± 0.01 (4)
RAC655	Susceptible	F	0.12 ± 0.03 (10)	0.53 ± 0.03* (12)	0.36 ± 0.09* (4)

^a^Plants for phenotyping were grown in a warm glasshouse but received no further treatment

^b^Plants were grown in a warm glasshouse and given a cool temperature shock of 7 days duration during the middle stages of grain ripening

^c^Plants were grown in a cool glasshouse with no further treatment

*Significantly different (alpha = *P* < 0.05, *n* = 4–10 untreated samples, *n* = 12 for cool temperature shock, *n* = 4 for cool temperature) from the means for other lines evaluated under the same conditions

### Populations derived by inter-crossing varieties differing in their genetic basis of resistance

**Fig. 1:**
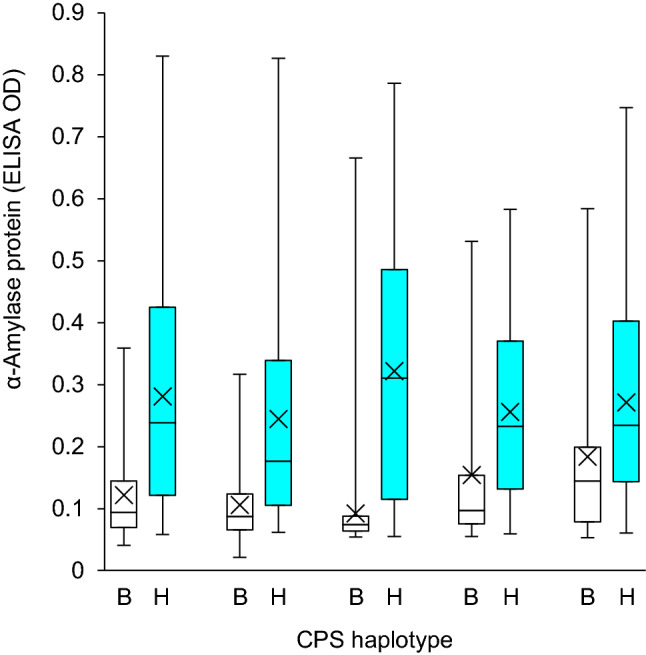
Phenotype (high-pI α-amylase protein content expressed as ELISA OD) distribution within homozygous LMA 7B genotype classes in the Halberd (LMA resistant)/Hartog (LMA resistant) population for one field and four glasshouse environments. Plants were sampled when mature and LMA phenotype was determined using ELISA. B and H refer to CPS haplotypes derived from Halberd (lighter boxes) and Hartog alleles (darker boxes), respectively. The horizontal line within each box represents the median whilst the mean is marked with a cross. The whiskers extend to the minimum and maximum observed values, respectively. Genotypic classes were significantly different within each experiment (*t* test, *P* < 0.05)

**Fig. 2:**
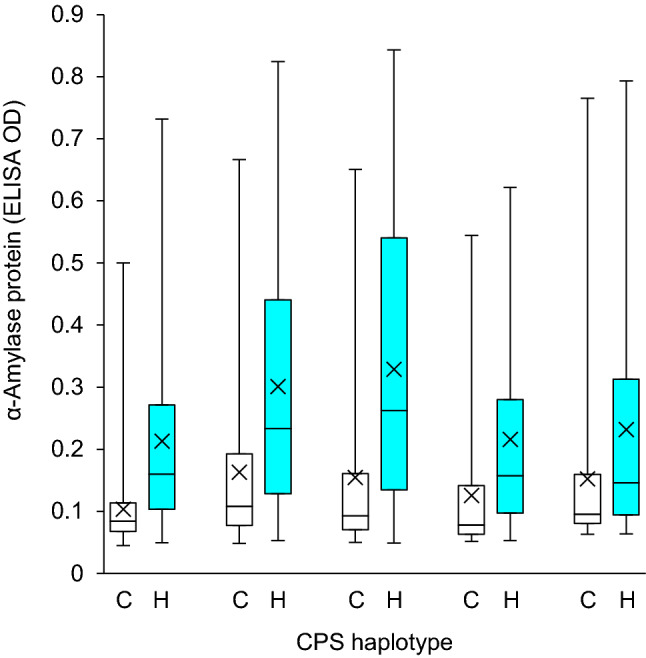
LMA phenotype (high-pI α-amylase protein content expressed as ELISA OD) distribution within homozygous genotype classes in the Maringa (LMA resistant)/Hartog (LMA resistant) population for one field and five glasshouse environments. Plants were sampled when mature and LMA phenotype was determined using ELISA. C and H refer to the CPS haplotypes derived from Maringa (lighter boxes) and Hartog (darker boxes), respectively. The horizontal line within each box represents the median whilst the mean is marked with a cross. The whiskers extend to the minimum and maximum observed values, respectively. Genotypic classes were significantly different within each experiment (*t* test, *P* < 0.05)

Based on genotyping of the marker AWW525 in the Halberd (LMA-resistant)/Hartog (LMA-resistant) and the markers wri847 and wri848 in the Maringa (LMA-resistant)/Hartog (LMA-resistant) population, each of the populations was separated into two sub-populations that carried 7B alleles from the respective parents. In each experiment, the mean phenotypic values [high-pI α-amylase protein content (ELISA OD)] for sub-populations that carried Hartog alleles at the 7B locus were significantly higher (*P* < 0.05) than for sub-populations with 7B alleles from Halberd or Maringa (Figs. 1, 2). The phenotype for each line in each of the experiments for each population is shown in Supplementary Tables S3 and S4. Within each sub-population and particularly in the sub-populations that carried the Hartog 7B alleles, considerable variation in LMA phenotype was observed, with values ranging from very resistant to very susceptible. This variation was similar to that observed in a collection of 55 varieties with the same CPS haplotype as Hartog (Derkx et al. 2021). Lines from each experiment and for each population were ranked based on their phenotype in each experiment. Lines in the lower (resistant) section of the distribution in one experiment maintained this phenotype across experiments. The same applied to lines in the upper (susceptible) sections of the distributions. For Halberd/Hartog, the mean phenotypes of the 20 lines at the resistant or the susceptible extremes of the distribution were 0.13 ± 0.03 and 0.30 ± 0.06 ELISA OD units, respectively. Similarly, for Maringa/Hartog, the means were 0.08 ± 0.01 and 0.41 ± 0.06 ELISA OD units, respectively. Whilst the phenotype distributions within the sub-populations with Halberd or Maringa 7B alleles were skewed towards resistance, in each case there were sufficient numbers of susceptible lines to demonstrate that the presence of these alleles did not guarantee a resistant phenotype.

### Populations derived from crosses between the LMA-resistant varieties Hartog and Janz and the LMA-susceptible line RAC655

To investigate the genetic basis of the difference in LMA resistance between the LMA-resistant varieties Hartog (CPS haplotype H) and Janz (CPS haplotype F) and the LMA-susceptible line RAC655 (CPS haplotype F), populations of RAC655/Hartog and Janz/RAC655 recombinant inbred lines were subjected to DArTseq genotyping-by-sequencing and QTL analysis.

**Fig. 3:**
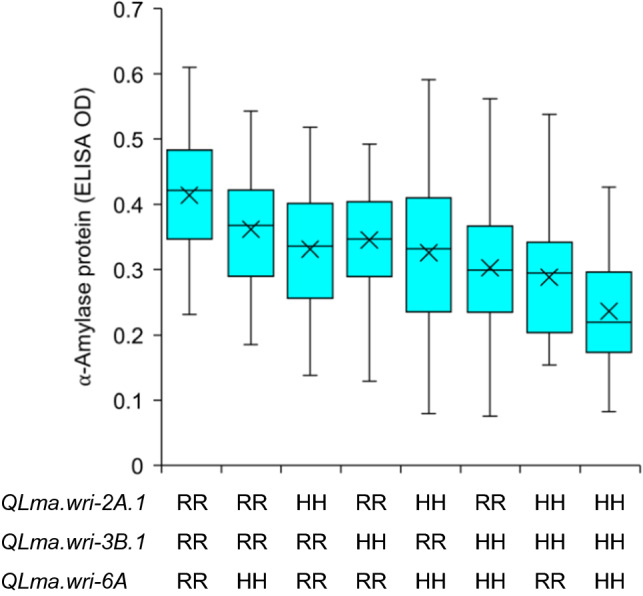
Distribution for LMA phenotypic values within the eight possible homozygous genotype classes for three QTL in RAC655 (LMA-susceptible)/Hartog (LMA-resistant), with lines classified based on the genotypes of the markers mapping at and/or flanking the estimated QTL positions reported in Table 2. RR and HH indicate homozygous RAC655 and Hartog genotypes at the respective QTL. The horizontal line within each box represents the median whilst the mean is marked with a cross. The whiskers extend to the minimum and maximum observed values, respectively

Based on DArTseq data for the RAC655/Hartog population, 3,777 SNPs were genetically mapped and minor QTL were detected on chromosomes 2A, 3B and 6A. At all three loci, the favourable alleles were from Hartog (Table 2). For each QTL position listed in Table 2, markers that mapped at or near that position can be found in Supplementary Table S5 together with marker name, genetic position, physical position (if known), reference tag sequence, SNP position within the tag sequence, identity and parental sources of the segregating nucleotides, and the genotypes for the mapping lines. Despite polymorphism at the CPS gene on chromosome 7B (Table 1), no QTL was detected on chromosome 7B. Among 343 lines whose genotypic data were used to construct the linkage map, 308 could be definitively classified as being homozygous for either RAC655 or Hartog alleles at all three estimated QTL positions. For these lines, comparison of phenotypic values among the eight possible homozygous genotypic classes shows the cumulative effect of favourable (Hartog) alleles in lowering high pI α-amylase protein ELISA OD values (Fig. 3). The phenotype for each line in each of the experiments is shown in Supplementary Table S6.

**Table 2:** Quantitative trait loci mapped for high pI α-amylase protein content in RAC655/Hartog, based on phenotypic means over glasshouse experiments conducted between 2016 and 2019, and in Janz/RAC655, based on phenotypic means over glasshouse experiments conducted in 2018 and 2019

Population	Chromosome	Linkage group^a^	Quantitative trait locus	Position on linkage group (cM)	LOD test statistic^b^	Additive effect^c^
RAC655/Hartog	2A	2A	*QLma.wri-2A.1*	88.53	7.5	0.033
	3B	3B	*QLma.wri-3B.1*	48.89	7.5	0.035
	6A	6A	*QLma.wri-6A*	110.53	3.7	0.024
Janz/RAC655	1B	1B1	*QLma.wri-1B*	25.00	5.4	− 0.039
	2A	2A2	*QLma.wri-2A.2*	126.19	5.4	0.041
	2B	2B2	*QLma.wri-2B*	139.09	15.3	0.062
	3A	3A1	*QLma.wri-3A.1*	83.35	4.0	0.038
	3A	3A2	*QLma.wri-3A.2*	165.11	9.4	0.054
	3B	3B1	*QLma.wri-3B.2*	259.54	7.5	0.049
	4A	4A2	*QLma.wri-4A*	44.00	5.6	− 0.042
	7D	7D1	*QLma.wri-7D*	9.63	4.2	0.038

^a^For RAC655/Hartog, the genetic map for each of these chromosomes consisted of only one linkage group. For Janz/RAC655, the genetic map for each of these chromosomes consisted of more than one linkage group. Details of the genetic maps are given in Supplementary Tables S5 and S7 where the quantitative trait loci can be located using the position on linkage group (cM) shown in the table

^b^All LOD values shown are statistically significant (genome-wide $\alpha = 0.05$) according to thresholds set based on 10,000 permutations (3.35 for RAC655/Hartog and 3.33 for Janz/RAC655)

^c^Positive additive effects indicate that alleles from RAC655 are associated with higher phenotypic values (LMA susceptibility). Negative additive effects indicate that alleles from RAC655 are associated with lower phenotypic values (LMA resistance)

Based on DArTseq data for the Janz/RAC655 population, a linkage map was constructed with 4015 SNPs assigned to genetic positions on 19 chromosomes (Supplementary Table S7 that includes the same details as RAC655/Hartog in Table S5). Due to segregation distortion imposed by selection for tall stature, the chromosomes carrying *Rht-B1* (4B) and *Rht-D1* (4D) are not included in the map. Eight minor QTL were detected (Table 2) for the Janz/RAC655 population. At six of these (*QLma.wri-2A.2*, *QLma.wri-2B*, *QLma.wri-3A.1*, *QLma.wri*-3A.2, *QLma.wri-3B.2* and *QLma.wri-7D*), the favourable alleles were from Janz. At the other two (*QLma.wri-1B* and *QLma.wri-4A*), the favourable alleles were from RAC655.

**Fig. 4:**
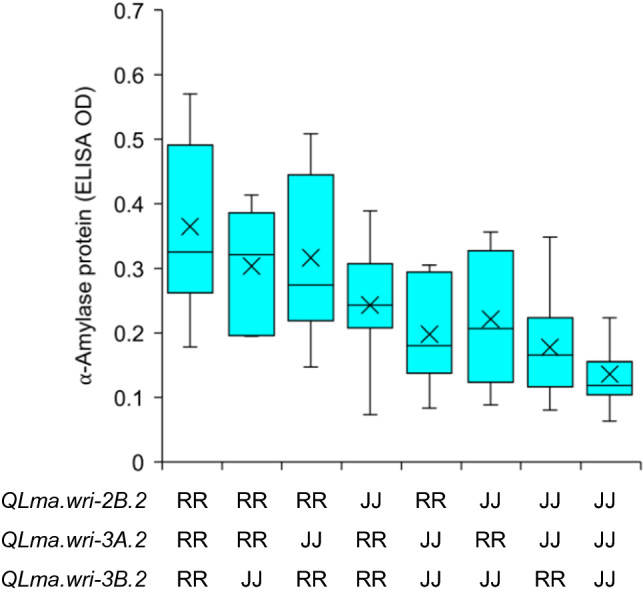
Distribution for LMA phenotypic values within the eight possible genotype classes for the three QTL with the greatest additive effects in Janz (LMA-resistant)/RAC655 (LMA-susceptible), with lines classified based on the genotypes of the markers mapping at and/or flanking the estimated QTL positions reported in Table 2. RR and JJ indicate homozygous RAC655 and Janz genotypes at the respective QTL. The horizontal line within each box represents the median whilst the mean is marked with a cross. The whiskers extend to the minimum and maximum observed values, respectively

With eight QTL, the number of expected genotypic classes (256) exceeds the number of lines genotyped in the Janz/RAC655 population. Accordingly, it is not feasible to graphically represent the phenotypic distributions for all classes. Instead, Fig. 4 shows a comparison for just the three QTL with the greatest additive effects (*QLma.wri-2B*, *QLma.wri-3A2* and *QLma.wri-3B.2*). Similar to what was seen for RAC655/Hartog (Fig. 3), there is a cumulative effect of favourable (Janz) alleles in lowering high pI α-amylase protein ELISA OD values. In this case, some of the phenotypic variation within each class would be due to segregation at the other five QTL. The phenotype for each line in each of the experiments is shown in Supplementary Table S8.

## Discussion

Crossing either of the LMA-resistant lines Halberd and Maringa, whose resistances are associated with a locus on chromosome 7B (Derkx et al. 2021), with the LMA-resistant line Hartog, whose resistance appears to be associated with loci located elsewhere on the wheat genome, resulted in a broad range of phenotypic variation. The results confirmed the strong effect of resistance alleles at the 7B LMA locus in Halberd and Maringa whilst also indicating that there must be additional QTL that contribute to determining the LMA phenotype. Lines at the extreme ends of the phenotype distributions in both populations maintained their phenotype across experiments, indicating that the underlying basis was genetic. Unfortunately, fixation of Halberd or Maringa alleles at the LMA 7B locus did not guarantee a resistant phenotype equal to that of the parental lines, indicating that a combination of these alleles with other loci may be required to achieve an effective low risk of LMA expression. At the time of conducting the studies involving the Halberd and Maringa populations, no loci associated with the resistance in Hartog had been identified. Minor loci had previously been identified on chromosome 3B in Halberd/Cranbrook (Mrva and Mares 2001a) and chromosomes 3A and 2D in Maringa/Spica (Derkx et al. 2021). Resistance alleles at these minor loci appeared to have a cumulative effect on LMA phenotype when combined with resistance alleles at the locus on chromosome 7B. Other studies have mapped loci on chromosomes 6B (two loci with seemingly opposite effects on phenotype) (Emebiri et al. 2010), 2D, 3A, 3B, 4B, 4D, 5D and 5B (Tan et al. 2010), 1A, 3B and 6B (glasshouse experiments; Liu et al. 2021b) and 3A, 7B and 7D (field experiments; Liu et al. 2021b).

The strategy employed in this study to dissect the genetic components of resistance in Hartog, as well as a similar resistant line, Janz, involved crossing these lines with a very susceptible parent, RAC655. All three parents had CPS haplotypes that do not appear to contribute to resistance (Derkx et al. 2021) and it was assumed that a very susceptible line such as RAC655 would be unlikely to carry alleles for resistance. Ultimately, the large RAC655/Hartog population proved difficult to phenotype for several reasons. Both parents are semi-dwarf lines that carry *Rht-D1b* and as a consequence, phenotyping required use of the cool temperature shock protocol described by Derkx and Mares (2020). The population is very large and whereas both parents reached anthesis at a similar time, the individual lines in the population exhibited significant variation for flowering time. The day to day variation in temperature in the glasshouse used to phenotype the RAC655/Hartog population meant that the temperature prior to the cool shock was potentially not consistent across all lines. In addition, Derkx and Mares (2020) had shown that wheat varieties vary according to the timing of their response to the cool temperature shock. Combined, these issues potentially introduced greater error in the phenotyping. Nevertheless, minor QTL with resistance alleles from Hartog were detected on chromosomes 2B, 3B and 6A. The loci had a cumulative effect on α-amylase protein content.

By contrast, Janz and RAC655 had the same 7B alleles but different semi-dwarfing genes. Without selection, four types of homozygous line would be expected among Janz/RAC655 progeny: tall lines with neither semi-dwarfing gene, *Rht-B1b* semi-dwarf lines, *Rht-D1b* semi-dwarf lines and double-dwarf lines with both *Rht-B1b* and *Rht-D1b*. Therefore, selection for tall stature was imposed during population development to provide a final Janz/RAC655 population comprised solely of tall lines that could be phenotyped for LMA without the need for a cool temperature shock and produce more consistent results. In this population, minor QTL were detected on chromosomes 2A, 2B, 3A, 3B and 7D (all with resistance derived from Janz) and on chromosomes 1B and 4A (with LMA resistance derived from RAC655, despite it being very susceptible). The loci had a cumulative effect on α-amylase protein content. Based on comparison between the RAC655/Hartog map of chromosome 2A with the Janz/RAC655 map of linkage group 2A3, the QTL *QLma.wri-2A.1* (RAC655/Hartog) and *QLma.wri-2A.2* (Janz/RAC655) are in similar positions. They may represent the same locus. Of seven co-segregating markers at the estimated position of *QLma.wri-2A.2*, six were also mapped for RAC655/Hartog: five within 5 cM distal to the estimated position of *QLma.wri-2A.1* and one at 3 cM proximal to that position.

These results pose a dilemma for wheat breeders who are attempting to develop new wheat varieties with a low risk of LMA expression. Some CPS gene sequence variants on chromosome 7B are associated with a low risk of LMA under most field conditions in Australia (Derkx et al. 2021). These can be readily tracked using molecular markers. Semi-dwarfing alleles at the *Rht-B1* and *Rht-D1* loci can also be tracked, but these may not provide sufficient resistance under all conditions. Improvements in LMA resistance beyond those conferred by alleles on 7B and at *Rht-B1* or *Rht-D1* may require pyramiding favourable alleles from various sources. While the results presented here confirm the existence of multiple QTL, and the SNP sequence information given in Supplementary Tables S4 and S6 could be used to develop marker assays, it is not known how diagnostic such assays would be across wheat breeding germplasm.

Ideally, the next logical step in the research would be to try and identify the genes underlying the resistance loci and develop markers based on gene sequence variants associated with resistance. This could be daunting. Forward genetics approaches (map-based cloning) would be limited given the relatively low effects of the individual resistance alleles, which would make it difficult to phenotypically classify recombinants. Further, *QLma.wri-2B* is in a large region of restricted genetic recombination (with 331 co-segregating markers at the QTL position), possibly corresponding with a previously reported introgression of a chromosome segment from *Triticum timopheevii* (Walkowiak et al. 2020).

Reverse genetics approaches to test hypotheses about candidate genes would be limited by the paucity of knowledge about the biochemical pathway that results in the coordinated synthesis of *α-Amy1* genes located on the group 6 chromosomes during LMA expression.

Further research is required to determine how many and what combination of QTL are required for an appropriately low level of risk in different environments, and piece together the steps that lead to the transcription of the *α-Amy1* genes and the synthesis of α-amylase proteins. A more detailed study of the effects of temperature on LMA and individual QTL effects would also be advantageous.

### *Author Contribution Statement*

DM, KM and AD conceived and designed the research and performed the phenotyping. DM developed the populations. JC performed genotyping. AD. AD and DEM conducted linkage mapping and QTL analysis. DM wrote the manuscript with input from the other authors. All authors read and approved the manuscript.

## Supplementary Information

Below is the link to the electronic supplementary material.

Supplementary file1 (PDF 1644 KB)

Supplementary file2 (XLSX 12 KB)

Supplementary file3 (XLSX 16 KB)

Supplementary file4 (XLSX 21 KB)

Supplementary file5 (XLSX 26 KB)

Supplementary file6 (XLSX 4739 KB)

Supplementary file7 (XLSX 30 KB)

Supplementary file8 (XLSX 3002 KB)

Supplementary file9 (XLSX 23 KB)

Supplementary file10 (PDF 445 KB)

Supplementary file11 (PDF 115 KB)

## Data Availability

All data generated or analysed during this study are included in this published article [and its supplementary information files].

## Abbreviations

CPS: *ent*-Copalyl diphosphate synthase
ELISA: Enzyme-linked immunosorbent assay
LMA: Late-maturity α-amylase
OD: Optical density
QTL: Quantitative trait locus
SNP: Single nucleotide polymorphism
