# Supplementary material for: Multiple loci with cumulative effects on late maturity α-amylase (LMA) in wheat
Source: Planta. 2023 Apr 11;257(5):96. doi: 10.1007/s00425-023-04131-1 (PMC10089986; doi:10.1007/s00425-023-04131-1)
Supplement: Supplementary file 1 — Supplementary file1 (PDF 1644 KB) [file 425_2023_4131_MOESM1_ESM.pdf]

Article: Multiple loci with cumulative effects on late maturity α-amylase (LMA) in wheat
Journal: Planta
Authors: Daryl Mares, Adinda Derkx, Diane E. Mather, Judy Cheong, Kolumbina Mrva
Corresponding author: Daryl Mares, The University of Adelaide,
daryl.mares@adelaide.edu.au

Fig. S1

a **b**

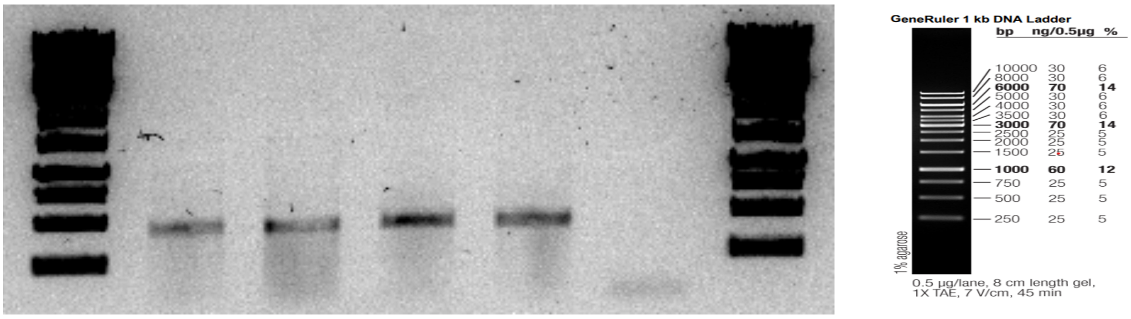

a Amplicons obtained from DNA samples of Halberd (second and third lanes) and Hartog (fourth and fifth lanes) using primers for the TaGAP housekeeping gene (F: TTCAACTCATTCCAAGCAGCA and R: CTGTAACCAAATGCCCTTG), all showing the expected product (469 bp), demonstrating the quality of the DNA samples. The sixth lane shows the result for a no-template control (no 469-bp amplicon), while the first and last lanes show results for Thermofisher Scientific GeneRuler 1-kb DNA Ladder **b** Amplicon sizes of products in Thermofisher Scientific GeneRuler 1-kb DNA Ladder

Fig. S2

a **b**

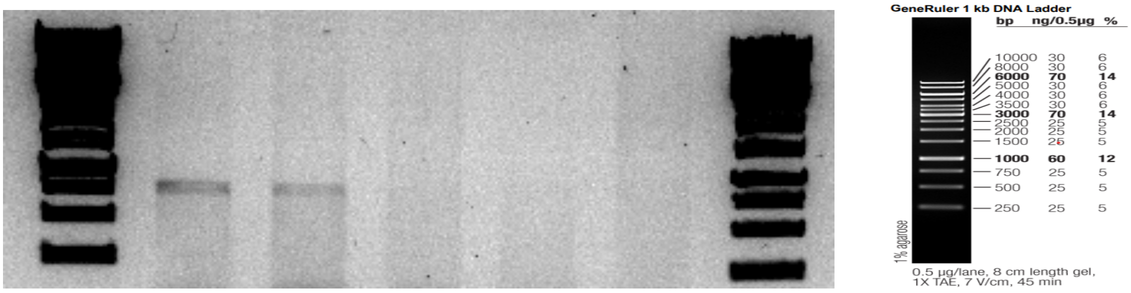

a Amplicons obtained from the same DNA samples of Halberd (second and third lanes) and Hartog (fourth and fifth lanes) as in Fig. S1, using primers for the marker AWW525 (F: GTTTTGGGGAGTGGTCCAGGTTCG and R: TATCTTACAAAGGACCAAGGATCGC), showing the presence of the expected product (748 bp) in Halberd and the absence of a corresponding product in Hartog and a no-template control (sixth lane). The first and last lanes show results for Thermofisher Scientific GeneRuler 1-kb DNA Ladder. **b** Amplicon sizes of products in Thermofisher Scientific GeneRuler 1-kb DNA Ladder

Fig. S3

a **b**

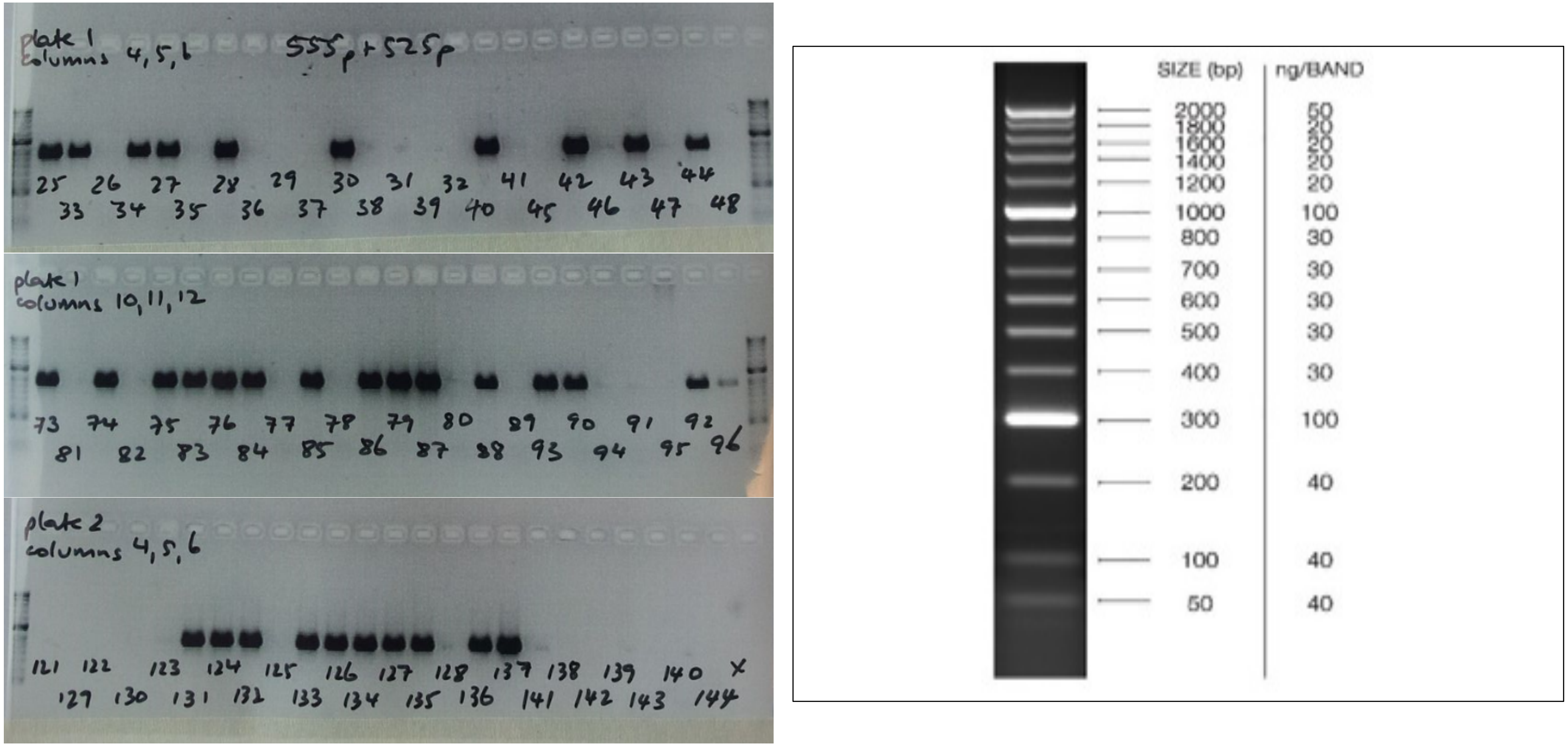

a Examples of gel images showing amplicons obtained from DNA samples of Halberd/Hartog lines using primers for the marker AWW525 (F: GTTTTGGGGAGTGGTCCAGGTTCG and R: TATCTTACAAAGGACCAAGGATCGC), showing clear presence-absence polymorphism for the expected showing the presence of the expected product (748 bp), with Bioline HyperLadder™ II (now called HyperLadder™ 50bp) in the first and last lanes of the first two gels and the first lane of the third gel. **b** Amplicon sizes of products in Bioline HyperLadder™ II
