# Supplementary material for: Multiple loci with cumulative effects on late maturity α-amylase (LMA) in wheat
Source: Planta. 2023 Apr 11;257(5):96. doi: 10.1007/s00425-023-04131-1 (PMC10089986; doi:10.1007/s00425-023-04131-1)
Supplement: Supplementary file 10 — Supplementary file10 (PDF 445 KB) [file 425_2023_4131_MOESM10_ESM.pdf]

Supplementary file S9 Mares DJ, Mrva K (1993) Late maturity -amylase in wheat. In: MK Walker-Simmons, JL Reid (eds). Preharvest sprouting in cereals 1992. American Association of Cereal Chemists, St Paul, Minnesota, USA, 178-184

LATE MATURITY α-AMYLASE IN WHEAT

D. J. Mares and K. Mrva
The University of Sydney, Plant Breeding Institute,
Narrabri, NSW 2390, Australia

SUMMARY

A number of wheat cultivars characterized by unacceptably high levels of α-amylase in ripe, non-sprouted grains have been identified in recent years. For a number of these cultivars the source of high α-amylase appears to be the Mexican cultivar Lerma 52 and its parent Mentana. Levels of α-amylase activity in ripe grain were influenced by the environment and genetic background. In particular, when grain ripened in a cooler, more humid glasshouse environment α-amylase production in reputed high amylase lines was further enhanced while in some cultivars, which maintained a low α-amylase profile similar to controls in the field, α-amylase production was stimulated sufficiently to allow the potential problem to be identified. Glasshouse culture thus appeared to provide a more reliable means of identifying cultivars prone to late maturity α-amylase and to give better discrimination between cultivars and within progeny derived from crosses involving a high amylase parent. Finally, further evidence is presented to support the hypothesis that late maturity α-amylase production is controlled by the endosperm tissues rather than the embryo.

INTRODUCTION

In the absence of sprouting some wheat cultivars produce α-amylase during the later stages of grain ripening sufficient to reduce

the falling number and amylograph peak viscosity to unacceptable levels. In some cultivars, *e.g.*, Huntsman (UK), this only occurs in some seasons, apparently in response to a specific set of environmental conditions (Gale and Lenton, 1987). Other cultivars by contrast *e.g.*, Spica (Australia) and Lerma 52 (Mexico), produce late maturity α-amylase under a wide range, if not all, growing conditions (Mares and Gale, 1990), although the effect is most dramatic in a cooler, humid ripening environment. This phenomenon has been examined in some detail using the cultivar Spica (Mares and Gale, 1990). In this cultivar synthesis of late maturity α-amylase begins approximately 40 days post anthesis, involves α-amylase isozymes controlled by genes on the group 6 chromosomes (*i.e.*, high pI isozymes typical of the early stages of germination), is controlled by a single recessive gene and is modulated by the environment and the genetic background. Enzyme was distributed throughout the length of the grain, thus contrasting with germinated grain where there is a marked gradient from the embryo to the distal end.

Recently in Australia new sources of late maturity α-amylase were identified in a number of breeding programs. For two breeding programs this has meant withdrawing potential new cultivars prior to, or following, release and necessitated a critical evaluation of the programs' germplasm base.

The aims of this investigation were to catalogue the pedigrees of genotypes exhibiting late maturity α-amylase, to compare the production of amylase in the field and the glasshouse, and to examine segregation for α-amylase in reciprocal crosses.

MATERIALS AND METHODS

Wheat cultivars were grown in the field at Narrabri until flowering at which time some plants were transplanted to a glasshouse (temperature range 16-25°C). Plants in the field ripened in a hot, dry environment in contrast to the cooler, more humid glasshouse environment. At harvest ripeness (12% moisture) grain was harvested from all plants for falling number and α-amylase determinations. α-Amylase was determined on single grains using a modification of the method of Barnes and Blakeney (1974) and relative activities per grain expressed as OD (optical density) units.

RESULTS

Sources of late maturity α-amylase

The most important source of late maturity α-amylase in Australian breeding programs would appear to be the cultivar Mentana (Table I). This cultivar appears in the parentage of Lerma

TABLE I
Falling Numbers (FN) and Pedigrees of Wheat Cultivars
Grown at Narrabri in Northern NSW

Cultivars and Pedigree	Origin	FN Range (sec.)
Sunco<br>(control cultivar)	University<br>of Sydney	390 - 450
Mentana		250 - 375
Lerma 52<br>(Mentana*3/Kenya 324	Mexico	185 - 250
Gamenya<br>(Gabo*6/Mentana W1124//<br>Gabo*2/Kenya 117A W1347)	Univ. Sydney	240 - 380
Veery lines<br>(Kavkaz/Buho "S"//Kal/BB where Buho "S" =<br>SR/3/Lr64/Inia 66/Inia 66/BB)	Mexico	
1		185 - 220
5		230 - 280
7		145 - 220
Veery 2,3,4,6,8 and 9		350 - 428
Inia 66<br>(Lr64/Son 64)	Mexico	380 - 420
Reeves<br>Bodallin//Gamenya/Inia 66)	W. Aust.	240 - 310
IW911 (Bolsena select.)<br>WE/3/Lr64/Inia66//Inia 66/BB/4/7C//TOB/CNO"S")	W. Aust.	242 - 315
77W 888<br>(Centrifen/Gamenya//Gamenya/Jacup)	W. Aust.	222 - 291
77W 884<br>(Centrifen/Gamenya//Gamenya/Jacup	W. Aust.	231 - 326
79W 793<br>(Wialki SIB//Lance/Eradu)<br>Eradu = Ciano/Gamenya	W. Aust.	175 - 220
Spica<br>(Three Seas/Kamburica//Pusa 4/Flora)	Aust.	189 - 235
Suneca<br>(Spica/Ciano 67/Amber<br>mut. Sonora)	University<br>of Sydney	375 - 410
BD 159<br>(Jabiru/Millewa//Millewa/TM56	Victoria	430 - 480

52 (used to develop Inia 66 and the Veery lines, Mexico) and Gamenya (an early University of Sydney cultivar, used extensively in Western Australia). The source of high amylase in Spica has not been ascertained. This latter cultivar was used in the development of Suneca, a high quality semi-dwarf wheat in which the high amylase gene appears to be held in check by some unknown factor.

A number of Western Australian lines gave low falling numbers in nurseries at Narrabri under conditions, which where not conducive to sprouting (Table I), and where cultivars recognized as being susceptible to sprouting maintained high falling numbers. Whilst Inia 66 and Gamenya have been implicated in the transfer of late maturity α-amylase, these lines commonly had high falling numbers in the Narrabri environment.

Similarly, BD159 invariably had a high falling number when grown in northern NSW. This cultivar exhibited a wide range of falling numbers when grown in Victoria (Table II, J. Panozzo, unpublished data), but its pedigree gives no indication as to the source of high α-amylase. BD159 has shown a high degree of variability or environmental sensitivity in marked contrast to cultivars such as Spica and Lerma 52.

Comparison of field-ripened and glasshouse ripened samples

α-Amylase activities in grain of control cultivars were similar and low in both ripening environments (Fig. 1a). Cultivars such as Spica and Lerma had high grain α-amylase levels in both environments, however, levels were highest in glasshouse-ripened samples (Fig. 1b).

TABLE II
Range of Falling Numbers and Amylograph Peak Heights
of Samples of BD159 and Meering,
Grown at 10 Sites in Victoria in 1989
(data supplied by J. Panozzo)

Cultivar	Amylograph Peak	Falling Number
Meering	450 - 860	375 - 464
BD159	< 100 - 760	72 - 499

By contrast, for the cultivar BD159, α-amylase levels were generally low and similar to control cultivars in the field material but were elevated, relative to controls in the glasshouse (Fig. 1c).

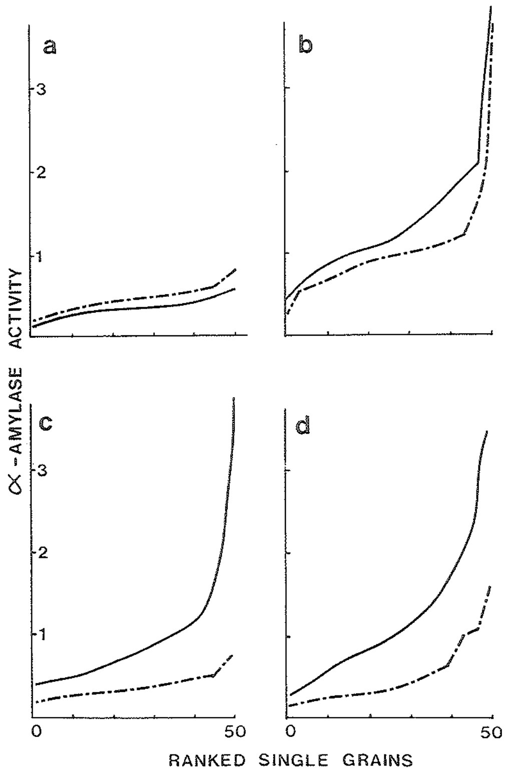

Fig. 1. Profiles of α-amylase activities in grains from wheat cultivars or F_1 hybrid plants ripened in the field (-·-) or in a glasshouse (—).

Activities in 50 single grains per sample were ranked in ascending order.
a. Janz (control - low α-amylase)
b. Spica (high α-amylase)
c. BD159 (variable α-amylase)
d. Chinese Spring (low α-amylase) X Spica (high α-amylase) F_2 grains.

The distribution of α-amylase was also examined in F_2 grains on F_1 plants from the cross Chinese Spring (low α-amylase) x Spica (high α-amylase) (Fig. 1d). Again α-amylase levels in individual grains were higher in the glasshouse-ripened sample and there was greater differentiation between high α-amylase and low α-amylase grains.

Distribution of α-amylase in F_1 grains of reciprocal crosses between Chinese Spring and Spica, and Chinese Spring and Lerma 52

Mean values of α-amylase in F_1 grains were similar to those of the low α-amylase parent, Chinese Spring, however, there was a consistent trend towards higher mean α-amylase where the high α-amylase parent was the female (Table III). Analysis of variance indicated that the difference was significant (P=0.01) for the reciprocal crosses involving Chinese Spring and Lerma only.

TABLE III
Mean α-Amylase Levels in F_1 Grains of Reciprocal Crosses Compared with Parent Cultivars

Cultivar or Cross ♀ ♂	Mean α-Amylase[1] (relative units)	Standard Deviation	F
Cs[2] x Sp[3]	0.26	0.13	2.92
Sp x Cs	0.33	0.12	
Cs x L[4]	0.16	0.04	16.99**
L x Cs	0.22	0.07	
Cs	0.24	0.07	35.13**
Sp	>2.1	0.67	
L	1.15	0.94	

[1] α-amylase in 30 grains of each cross or parent
[2] Chinese Spring
[3] Spica
[4] Lerma 52

DISCUSSION

Late maturity α-amylase production represents a serious problem to breeders, grain receival agents and marketing authorities. The safest course of action would be to avoid the use of high amylase germplasm, however, in some instances these lines have a number of desirable agronomic or quality attributes. Selection within breeding populations for low α-amylase is made difficult by the recessive nature of the character, the influence of the environment, and the genetic background (*e.g.*, expression of high α-amylase gene is very low in warmer environments). In the cases of genotypes showing extreme sensitivity to the environment, selection may not be possible in many environments.

Glasshouse-ripened samples exhibited higher levels of α-amylase, gave better discrimination between high and low types, and elicited a response in some cultivars that maintained low α-amylase in the field. Whereas this technique offers some hope for breeders, there is nevertheless an urgent need to develop screening techniques (*e.g.*, biochemical or molecular markers) capable of identifying grains homozygous for low α-amylase.

The observed trend, within F_1 grains, for α-amylase to be slightly higher when the high amylase parent was used as the maternal parent is consistent with control being exercised by the triploid endosperm and aleurone rather than the diploid embryo. This suggestion is supported by previous investigations (Mares and Gale, 1990) which noted that enzyme detected in scraped aleurone tissue was distributed along the length of the grain, rather than concentrated near the embryo as in germinated grains.

REFERENCES

Barnes, W. C. and Blakeney, A. B. 1974. Determination of cereal α-amylase using a commercially available dye labelled substrate. Staerke 26:193-197.

Gale, M. D. and Lenton, J. R. 1987. Pre-harvest sprouting in wheat - a complex genetic and physiological problem affecting bread making quality of UK wheats. In: Aspects of Applied Biology. The Association of Applied Biologists, UK, 15:115-124.

Mares, D. J. and Gale, M. D. 1990. Control of alpha-amylase synthesis in wheat grains. In: Proceedings of the Fifth International Symposium on Pre-Harvest Sprouting in Cereals. K. Ringlund, E. Mosleth, and D. J. Mares, eds. Westview Press, Boulder, CO, USA, pp. 183-194.

THE ROLE OF A NOVEL EMBRYO-CAVITY WAX IN PREVENTING SPROUTING

A. D. Evers and J. Kratochvil*
Flour Milling and Baking Research Association
Chorleywood, Hertfordshire WD3 5SH, England

SUMMARY

Wax crystals were found in the embryo cavity of long-stored red wheat varieties, but none was found in white varieties stored for the same time. It is hypothesized that the wax from which the crystals developed may have played a role in controlling the entry of water into the grain during the critical post-ripening stage when pre-harvest sprouting is a hazard. Should the hypothesis be proved the wax characteristics may provide a marker for sprout resistance in early generation stocks. Wax has been dissected from the old samples of red grain, and its composition determined, for comparison with waxes extracted from fresher grains. The waxes comprise about 200 components. Some systematic differences exist between sprout-resistant and sprout-susceptible types.

INTRODUCTION

A chance observation was made under the scanning electron microscope, of tufts of needle-like structures in the embryo cavity of grains of the old English wheat variety Yeoman which had been stored for 25 years.

---

*Present address: Institute of Macromolecular Chemistry, Prague 6, 16206, Czechoslovakia

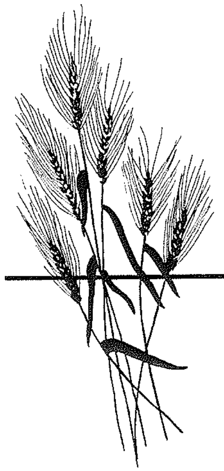

Pre-Harvest Sprouting in Cereals 1992

Edited by
M. K. Walker-Simmons
J. L. Ried

USDA-ARS
Washington State University
Pullman, Washington

American Association of Cereal Chemists
St. Paul, Minnesota U.S.A.
