# Supplementary material for: Multiple loci with cumulative effects on late maturity α-amylase (LMA) in wheat
Source: Planta. 2023 Apr 11;257(5):96. doi: 10.1007/s00425-023-04131-1 (PMC10089986; doi:10.1007/s00425-023-04131-1)
Supplement: Supplementary file 11 — Supplementary file11 (PDF 115 KB) [file 425_2023_4131_MOESM11_ESM.pdf]

Supplementary File S10

Mrva, K., Mares, D. and Cheong, J. (2008) Genetic mechanisms involved in late maturity α-amylase in wheat. In: R Appels, R Eastwood, E Lagudah, P Langridge, M MacKay, L McIntyre, P Sharp (Eds). Proceedings of the 11th International Wheat Genetics Symposium, Brisbane 2008. Sydney University Press pp 940-942

Genetic Mechanisms Involved in Late Maturity α-Amylase in Wheat

Mrva K[1], Mares DJ[1], Cheong J[2]

[1]School of Agriculture, Food and Wine, University of Adelaide, Waite Campus, Glen Osmond, SA 5064, [2]SARDI, GPO Box 397, Adelaide SA 5001, Australia

INTRODUCTION

Late maturity α-amylase (LMA) is a genetic disorder associated with premature synthesis of high pI α-amylase isozymes during the middle to later stages of grain development under normal growing conditions or, more commonly, when exposed to a cool temperature shock [1,2]. For the majority of LMA-prone genotypes, exposure to a significant temperature differential (i.e., a cool temperature shock), rather than cool temperature alone appears to be important for consistent and maximum expression of LMA [3]. High α-amylase activity is retained in the grain at harvest ripeness, resulting in low falling number and consequently a failure to meet receival standards. LMA in wheat is associated with QTL located on chromosomes 7B and 3B [4] that appear to be independently effective and additive. The level of expression is affected by a range of factors that include: genotype, environment and agronomy [2,5].

This aim of this study was to compare the levels of LMA expression in tall and semi-dwarf *Triticum aestivum* genotypes, Seri isolines in which 1B/1R and 7Ag/7D translocations were present or absent, *Triticum durum* genotypes and primary synthetic hexaploids.

MATERIALS AND METHODS

Non-LMA genotypes included Halberd (*rht*), Hartog (*Rht2*) and Krichauff (*Rht1*). LMA-prone genotypes included Spica (*rht*), Lerma52 (*rht*), Suneca (*Rht2* semi-dwarf with Spica in its pedigree), Cranbrook and BD159 (*Rht1* semidwarfs), Kennedy and Super Seri (*Rht1* semi-dwarfs derived from Seri but lacking the 1B/1R wheat/rye translocation), Seri (*Rht1* semi dwarf containing 1B/1R), ChuanMai18 (*Rht8* semi-dwarf) and Reeves (intermediate height and not containing *Rht1* or *Rht2*. R. Wilson pers comm.). 3 sets of Seri isolines with and without the 1B/1R and *7Ag*/7D translocations, were obtained from Cimmyt. Primary synthetic hexaploids were also developed in Cimmyt [6] whilst a doubled haploid population, Worrakatta/AUS29663 was obtained from Dr Wallwork, SARDI, Australia. Worrakatta is a non-LMA, *Rht1* cultivar and AUS29663 is a synthetic hexaploid with pedigree Ceta/*Ae tauschii* (895). Durum cultivars used in the development of some synthetic hexaploids were obtained from the Australian Winter Cereals Collection, Tamworth, Australia.

Plants were grown in field and glasshouse nurseries and midway through grain development a sample of tillers was subjected to a cool temperature shock [1] then transferred to a warm environment to complete ripening. The remaining tillers on plants in the field or glasshouse nurseries were allowed to ripen naturally. α-Amylase activity in the distal halves of grains was determined with high pI-specific ELISA according to Mrva and Mares [4] and data expressed as a percent of grains with activity greater than controls. Similarly, QTL analysis was as described by Mares *et al.* [7].

RESULTS

Data accumulated over several years consistently showed that whereas tall LMA-prone genotypes e.g. Spica (also Lerma52 - not shown) exhibited constitutive expression, most semi-dwarf genotypes required a cool temperature shock during mid-grain development for grain to develop high levels of α-amylase activity (Fig. 1). LMA-prone breeding lines and cultivars, Kennedy, Suneca, BD159, Cranbrook and Super Seri, which contained *Rht1* or *Rht2* had low α-amylase activity in untreated but elevated activity in treated samples. In marked contrast, Reeves, Seri and ChuanMai18 had high grain α-amylase activity in both treated and untreated samples. A number of Chinese wheat cultivars related to ChuanMai18 (data not shown) also gave high grain α-amylase activity without a cool temperature shock.

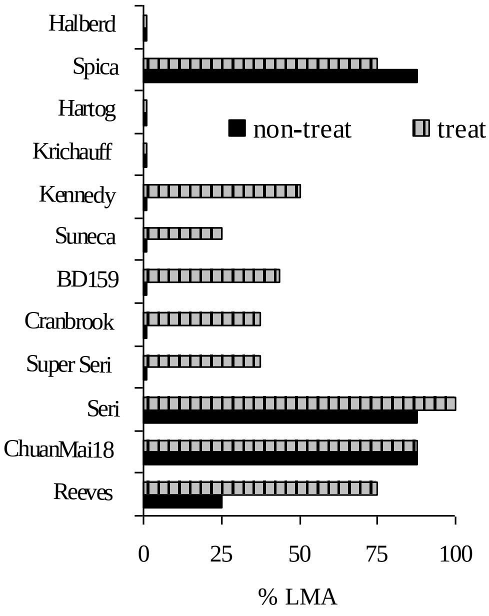

Figure 1. Percent grains with high α-amylase activity (% LMA) in samples from cool shock treated and untreated LMA-prone genotypes and non-LMA cultivars.

Non-LMA cultivars such as Halberd, Hartog and Krichauff maintained low α-amylase activity irrespective of the cool temperature treatment.

Preliminary results from a study of a Hartog/Seri RIL population suggested that the very high expression of LMA was associated with the presence of the 1B/1R translocation in combination with LMA QTL. This hypothesis was tested by comparing α-amylase activity in sets of Seri near-isogenic lines grown in the field and glasshouse and subjected to the cool temperature shock treatment. In isolines containing the 1B/1R translocation, the level of LMA was very high and similar to the recurrent parent, Seri (Fig. 2). By contrast, in the absence of this translocation levels of α-amylase activity were significantly reduced in both field and glasshouse material. The presence of the *7Ag*/7D translocation by comparison had little if any effect.

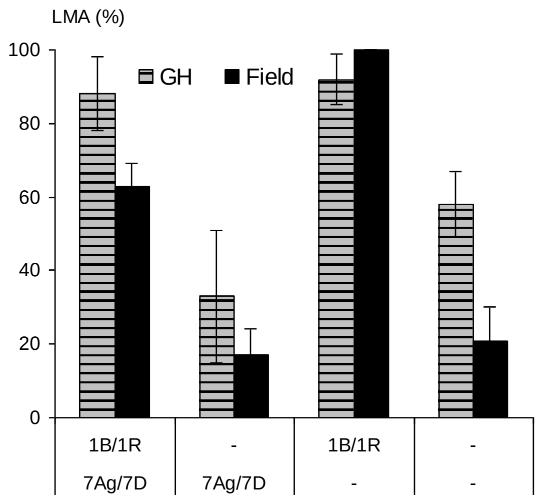

Figure 2. Comparison of LMA in near-isogenic lines of Seri grown in the field and a glasshouse (GH).

Over 300 primary synthetic hexaploid wheats have now been screened for LMA. More than 90% of the primary synthetics developed LMA during grain ripening and, of these about half had extreme levels of α-amylase normally only seen in wheats lacking semi-dwarfing genes [8]. A very high proportion of the primary synthetics were tall to very tall, despite the expected presence of *Rht1* from the durum parents, and when a subset of the tall synthetics was compared with and without a cool temperature shock there was no significant difference in grain α-amylase activity at ripeness.

Doubled haploid lines from Worrakatta/AUS29663 varied substantially for LMA phenotype, ranging from zero similar to Worrakatta to very high typical of AUS29663. Lines with very high expression contained the AUS29663 alleles at both the LMA 7B and 3B QTL or the AUS29663 allele at the LMA 7B QTL. Lines with Worrakatta alleles at both loci had phenotypes similar to that parent. In addition to the LMA QTL, a highly significant QTL coinciding with the marker for *Rht1* was identified (LOD = 4.6 and 7.1 in field and glasshouse environments respectively).

A sub-set of durum cultivars that have been used in the development of synthetic hexaploids, Yavaros79, Aconchi89, Snipes, Altar84, Croc S and Scooper were screened in summer nurseries in 2007/08. Preliminary data suggested that whereas Altar84 and Yavaros79 were very low, others such as Croc S were associated with extremely high levels of LMA. Interestingly, the LMA phenotype of primary synthetics derived from both Altar84 and Croc S ranged from zero or very low to extremely high. Similar patterns were observed in other groups of synthetics with a common durum parent.

DISCUSSION

Previous research has shown that GA insensitive semi-dwarfing genes such as *Rht1*, *Rht2* and *Rht3* reduce the level of LMA expression [9]. In addition, whereas tall LMA-prone wheat cultivars produce high levels of α-amylase activity in most if not all environments, LMA-prone semi-dwarfs appear to require a cool temperature shock to trigger synthesis of α-amylase. In the absence of this shock, field grown semi-dwarf LMA-prone genotypes have a non-LMA phenotype.

The results suggest that GA insensitive/ semi-dwarfing genes not only reduce the level of expression but are also associated with a change from a constitutive pattern of expression to one the requires a cool temperature shock. The apparent constitutive pattern of LMA expression in some *Rht8* semi-dwarf genotypes where reduced height is attributed to mechanisms other than GA insensitivity, is consistent with this conclusion.

The change from constitutive to cool temperature shock requiring was reversed by the presence of the 1B/1R wheat/rye translocation but not *7Ag*/7D. The mechanism associated with the effect of 1B/1R remains unclear.

Synthetics showed extreme levels of LMA, presenting a limitation to their widespread use in breeding programs. The LMA phenotype appeared to be associated with the same QTL, 7B and 3B, identified in conventional bread wheats and the extreme constitutive expression can be explained, at least in part, by the absence of *Rht1*. The association of LMA in synthetics with QTL located on chromosomes 7B and 3B indicate that the trait is contributed by the durum parent even though in some instances the durum parent did not appear to have LMA. In addition, the wide variation in LMA phenotype within groups of synthetics hexaploids with the same durum parent suggests that other unidentified mechanisms may be involved.

ACKNOWLEDGEMENTS

The authors acknowledge the support of the Grains Research and Development Corporation.

REFERENCES

1. Mrva,K. and Mares,D.J. (2001) Induction of late maturity α-amylase in wheat by cool temperature. Australian Journal of Agricultural Research 52, 477-484

2. Mares, D.J. and Mrva, K. (2008) Late-maturity α-amylase: low falling number in wheat in the absence of preharvest sprouting. Journal of Cereal Science 47: 6-17

3. Mrva, K and Mares, D.J. (2007) Influence of temperature on the expression of late maturity α-amylase. Cereals 2006.Proceedings of the 56th Australian Cereal Chemistry Conference and Indian Ocean Rim Conference. C.L. Blanchard, V.A. Solar and G.B. Crosbie (eds) pp 21-24.

4. Mrva,K. and Mares,D.J. (2001) Quantitative trait locus analysis of late maturity α-amylase in wheat using the doubled haploid population Cranbrook x Halberd. Australian Journal Agricultural Research 52, 1267-1273.

5. Lunn, G.D., Major, B.J., Kettlewell, P.S., Scott, R.K., 2001. Mechanisms leading to excess alpha-amylase activity in wheat (*Triticum aestivum*,L.) grain in the UK. Journal of Cereal Science 33, 313–329.

6. Mujeeb-Kazi, A, Rosas V & Roldan S (1996) Conservation of the genetic variation of *Triticum tauschii* (Coss.) Schmalh. *(Aegilops squarrosa* auct. non L.) in synthetic hexaploid wheats (T. *turgidum*L. s.lat, x T. *tauschii;* 2n = 6x = 42, AABBDD) and its potential utilization for wheat improvement. Genetic Resources and Crop Evolution 43: 129-134.

7. Mares,D.J., Mrva,K., Cheong,J., Williams,K. , Watson,B., Storlie,E., Sutherland,M., Zou,Y. (2005) A QTL located on chromosome 4A associated with dormancy in white- and red-grained wheats of diverse origin. Theoretical and Applied Genetics 111(7): 1357-1364.

8. Mares, D.J. and Mrva, K. (2008) Genetic variation for quality traits in synthetic wheat germplasm. Australian Journal of Agricultural Research 59: 406-412.

9. Mrva,K. and Mares,D.J.(1996). Expression of late maturity α-amylase in wheat containing gibberellic acid insensitivity genes. Euphytica 88, 68-76
